# Decreased signalling of EphA4 improves functional performance and motor neuron survival in the SOD1^G93A^ ALS mouse model

**DOI:** 10.1038/s41598-018-29845-1

**Published:** 2018-07-30

**Authors:** J. Zhao, L. T. Cooper, A. W. Boyd, P. F. Bartlett

**Affiliations:** 1 Queensland Brain Institute, University of Queensland, Brisbane, Queensland Australia; 2 Queensland Institute of Medical Research, Brisbane, Queensland Australia

## Abstract

Amyotrophic lateral sclerosis (ALS) is an untreatable, progressive, neurodegenerative disease specifically affecting motor neurons. Recently, the tyrosine kinase receptor EphA4 was directly implicated in ALS disease progression. We report that a long-lived mutated form of the EphA4 antagonist EphA4-Fc (mutEphA4-Fc), which blocks EphA4 binding to its ligands and inhibits its function, significantly improved functional performance in SOD1^G93A^ ALS model mice, as assessed by rotarod and hind-limb grip strength tests. Further, heterozygous motor neuron-specific *EphA4* gene deletion in SOD1^G93A^ mice promoted significant improvement in functional performance during the disease course and a delay in disease onset relative to control mice. Importantly, mice in the heterozygous deletion group showed significantly improved survival of motor neurons and architecture of endplates of neuromuscular junctions compared with control and homozygous *EphA4*-deletion groups. Our novel results show that EphA4 signalling directly regulates motor neuron survival and that mutEphA4-Fc is a promising therapeutic candidate to slow disease progression in ALS.

## Introduction

EphA4 is a tyrosine kinase receptor encoded by the *EphA4* gene, which belongs to the Eph receptor family. Previous studies have shown that EphA4 is involved in commissure formation within the forebrain, axonal guidance in the corticospinal tract^1^, regulation of the central pattern generator that provides normal locomotor function^2^ and axonal regeneration following spinal cord injury^3–5^. Recently, several reports have implicated EphA4 as a disease modifier in amyotrophic lateral sclerosis (ALS)^6–8^ and indicated that either genetic or pharmacological inhibitors of EphA4 would reduce disease progression of ALS; however, the potential mechanism underlying this novel effect of EphA4 is still unclear.

Previously, we successfully produced an EphA4 receptor antagonist, EphA4-Fc, which is a soluble fusion protein combining the extracellular domain of wildtype EphA4 with an IgG Fc fragment. EphA4-Fc blocks the function of EphA4, resulting in significant improvement in function in rodents after spinal cord injury^3,5^. However, this wildtype EphA4-Fc has a relatively short half-life of less than 48 hrs^5^, limiting its therapeutic potential. Recently, human EphA4-Fc was mutated by our group using a novel glycoengineering method to greatly enhance its half-life without affecting its other characteristics^9^, enabling it to be developed as a potential treatment for ALS and other diseases where long-term treatment would be required. The aims of this study were to test whether such a potential therapeutic reagent would exert a protective effect on ALS progression in a commonly used model of ALS, the SOD1^G93A^ mouse model, and to determine how EphA4 modifies the progression of ALS.

## Methods and Results

We first fused the extracellular domain of mouse EphA4 (amino acid 1–546 of NP_031962.22) with the mouse Fc domain of IgG1 (amino acid 239–460 of CAD32497.1), according to previously described^3^. Three glycosylation sites in the mouse EphA4 ectodomain, N235, N340 and N408, were then site-directly mutated (mutEphA4-Fc), which are same with human mutant EphA4-Fc described above^9^. The mutEphA4-Fc was cloned into expression vector pcDNA3.1 (Invitrogen, Life Technologies), and the nucleotide sequence of the construct was verified by Sanger Sequencing. Expression vectors were transfected in to HEK-293T cells, and large-scale preparations were performed using QIAGEN Plasmid Giga Kits according to the manufacturer’s instructions. An aliquot of the mutEphA4-Fc was subsequently used to assess the ligand-binding activity and pharmacokinetics. As expected, mutEphA4-Fc exhibited comparable binding to the ephrin A5 ligand as wildtype mouse EphA4-Fc (see Supplementary Fig. 1A) but, as with the modified human protein, it had a much longer *in vivo* half-life (see Supplementary Fig. 1B).

**Figure 1:**
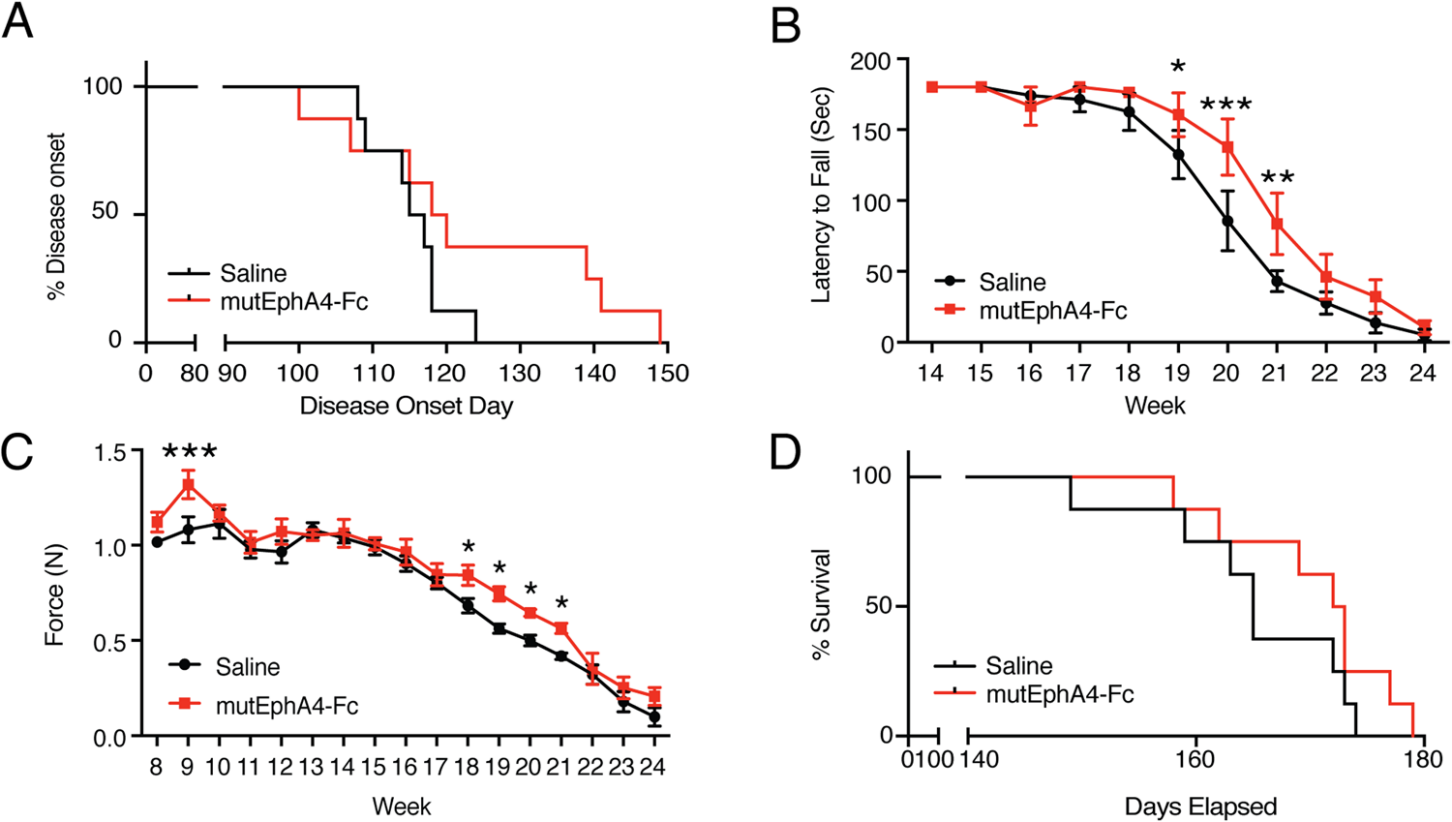
Effect of mutEphA4-Fc treatment on SOD1^G93A^ mice in functional tests and disease onset. (**A**) Kaplan Meier plot of ages (in days) of mutEphA4-Fc treated and saline control groups, in which the animal reached its peak body weight. The mean disease onset in the mutEphA4-Fc-treated group was delayed by about one week, compared with that in the saline control group; however, this difference was not statistically significance (log-rank test; *p* = 0.15). (**B**) Rotarod test values for mutEphA4-Fc treated and saline control groups show better performance of the mutEphA4-Fc treatment group from week 17 to week 23, compared with the control group (n = 8 per group; two-way ANOVA with Fisher’s LSD test at each age; *p < 0.05; **p < 0.01; ***p < 0.001). (**C**) Hind-limb grip strength; significant improvements in grip strength are seen in mutEphA4-Fc-treated SOD1^G93A^ mice versus saline-treated mice at 9 weeks and 18–21 weeks of age. (two-way ANOVA with Fisher’s LSD test at each age; *p < 0.05; ***p < 0.001). (**D**) Kaplan Meier plot of survival time (in days) of mutEphA4-Fc-treated and saline control groups. The mutEphA4-Fc treatment group displayed a longer median survival time compared with the control group; however, this difference was not statistically significant. (log-rank test; *p* = 0.19). Data are expressed as mean ± SEM; n = 8 per group.

Based on previous experiments in spinal cord injury models in which the minimal effective dose of EphA4-Fc was 10–20 mg/kg^3^, in this study a dose of 20 mg/kg was used to achieve the best potential therapeutic effect. Beginning at 5 weeks of age, all SOD1^G93A^ mice received intraperitoneal injections of either 20 mg/kg mutEphA4-Fc or an equal volume of saline (8 randomly selected mice per group), three times per week for the first 4 weeks to achieve a maximal cumulative dose, and then twice per week thereafter. These treatments were administered until the experimental end-point, which was determined for each mouse by observation of any of the following signs: loss of the righting reflex (unable to right within 30 seconds of being placed on their back), excessive weight loss (greater than 20% of their highest body weight), or complete paralysis of any hind-limb rendering the animal incapable of reaching food and water, at which point the mice were euthanised. This criteria for euthanasia has been previously described^10^. All SOD1^G93A^ mice from treatment and saline control groups had the same changes in body weight during disease progression; they gained weight at first but gradually lost weight until the end-point. We also compared disease onset in each group, which was retrospectively defined as the age at which the animal reached its peak body weight, according to previous described^11^. The mean disease onset in the mutEphA4-Fc-treated group was 123 days, which was a delay of one week, compared with that in the saline control group (mutEphA4-Fc group = 123.6 ± 6.18 days; saline group = 115.4 ± 1.83 days); however, this difference did not reach statistical significance (log-rank test for Kaplan-Meier survival plots; *p* = 0.15) (Fig. 1A). It was worth noting that five out of eight mice in the mutEphA4-Fc-treated group showed delayed disease onset, compared to the mean disease onset of the saline control group. Moreover, three of them showed delayed disease onset by about 3 weeks.

We next assessed whether mutEphA4-Fc would improve functional performance in SOD1^G93A^ mice, as assessed by rotarod and hind-limb grip strength tests, according to previously described^12,13^. Motor coordination in the mice was monitored from 8 weeks of age using an accelerating rotarod apparatus. The speed of the rod rotation was gradually increased from 2 to 20 rpm/min over 48 seconds. Each mouse was given three attempts and the longest latency to fall was recorded; 180 seconds was chosen as the cut-off time. In this test, a decline was initially detected at week 16 in both mutEphA4-Fc-treated and control groups. The SOD1^G93A^ mice receiving the mutEphA4-Fc treatment maintained better performance in this test than control SOD1^G93A^ mice from week 17 to week 23 and the differences at weeks 19–21 were statistically significant (Two-way ANOVA, followed by Fisher’s LSD test, *F*_(16,224)_ = 1.49; week 19, *p* = 0.0499; week 20, *p* = 0.0003; week 21, *p* = 0.0049) (Fig. 1B). In line with this result, mutEphA4-Fc-treated SOD1^G93A^ mice also showed significantly better maintenance of hind-limb grip strength at 9 weeks and 18–21 weeks of age, compared with the vehicle control group (Two-way ANOVA, followed by Fisher’s LSD test, *F*_(16,224)_ = 1.067; week 9, *p* = 0.0009; week 18, *p* = 0.0238; week 19, *p* = 0.01; week 20, *p* = 0.0403; week 21, *p* = 0.0386) (Fig. 1C). The mutEphA4-Fc treatment group also displayed a longer median survival time compared with the control group (mutEphA4-Fc group = 172.5 days (158–179); saline group = 165 days (149–174); log-rank test; *p* = 0.19) (Fig. 1D). Although this difference was not statistically significant, a similar difference was reported using the KYL EphA4-blocking peptide administered intracerebroventricularly^6^.

One of the pathological hallmarks of ALS is progressive loss of upper and lower motor neurons, and various factors have been reported to contribute to the motor neuron loss associated with ALS, including neurotoxicity, abnormal RNA processing, mitochondrial dysfunction, aberrant axonal transport and inflammation (reviewed in^14^). We therefore next investigated whether EphA4 regulates ALS pathogenesis by affecting motor neuron survival/loss. A previous study reported that homozygous deletion of EphA4 in SOD1^G93A^ mice generated animals that were not fertile or viable, suggesting that EphA4 plays a critical role in the development of the central nervous system (CNS) in this mouse model^6^. To further address the relationship between EphA4 and motor neuron survival, we produced SOD1^G93A^ mice with specific *EphA4* gene deletion in choline acetyltransferase (ChAT)-expressing cells. EphA4^flox/flox^ mice have a conditional allele of EphA4, with exon 3 flanked by two LoxP sites^15^, and when EphA4^flox/flox^ mice are crossed with ChAT-Cre^KI/KI^ mice^16^, Cre-mediated excision of exon 3 and splicing of exon 2 to exon 4 causes a frameshift in the downstream sequence of *EphA4*, resulting in specific loss of EphA4 expression in ChAT-expressing cells. Significant ChAT expression in cholinergic neurons is detected from postnatal day 5 (P5) and is sustained throughout adulthood^16,17^, and both upper and lower motor neurons are the cholinergic neurons that gradually degenerate in ALS. Therefore, the *EphA4* gene was functional during the embryonic period in this mouse model, allowing normal development, with specific deletion of EphA4 expression in motor neurons after P5, thus providing an optimal model to investigate the effect of EphA4 on motor neurons in ALS.

**Figure 2:**
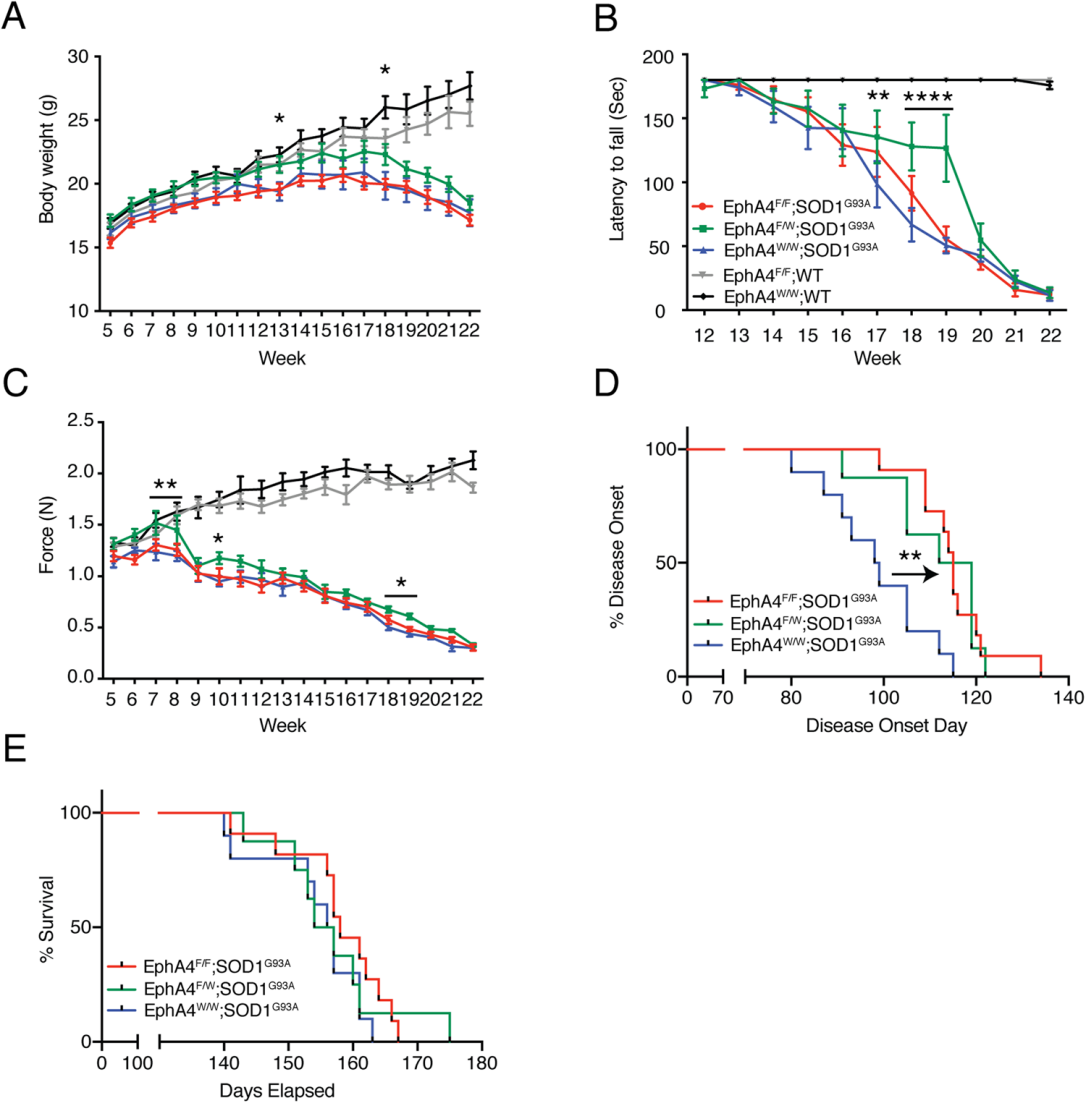
Conditional knockout of the EphA4 gene in SOD1^G93A^ mice improved functional performance, delayed disease onset, but did not affect survival. (**A**) SOD1^G93A^ mice with heterozygous deletion of EphA4 in ChAT-positive motor neurons were heavier than homozygous deletion mice or normal SOD1^G93A^ mice. Wild-type mice with or without the EphA4 gene exhibited gradual increases in body weight with no significant difference between these two groups. (**B**,**C**) Changes over time in (**B**) rotarod test values and (**C**) hind-limb grip strength. EphA4^F/W^; SOD1^G93A^ mice showed better function in both tests compared with EphA4^W/W^; SOD1^G93A^ mice. In the rotarod test, EphA4^F/F^; WT and EphA4^W/W^; WT mice continued to perform at near optimal levels. Additionally, the hind-limb grip strength of EphA4^F/F^; WT and EphA4^W/W^; WT mice steadily increased with time until the end-point (two-way ANOVA with Fisher’s LSD test at each age; *p < 0.05; **p < 0.01; ***p < 0.001; ****p < 0.0001). (**D**,**E**) Kaplan Meier pots of (**D**) disease onset and (**E**) survival time in the three SOD1^G93A^ groups. (**D**) Both homozygous and heterozygous deletion of *EphA4* in ChAT-positive motor neurons in SOD1^G93A^ mice delayed disease onset relative to normal SOD1^G93A^ mice (log-rank test; **p < 0.01). (**E**) Survival times of each group are not significantly different (log-rank test; *p* = 0.47). Data are expressed as mean ± SEM; *n* = 8–11 mice per group.

We closely monitored and compared body weight, functional performance and motor neuron survival in the spinal cord lumbar enlargement between experimental groups, including EphA4^flox/flox^ × ChAT-Cre^KI/WT^ × SOD1^G93A^ mice (EphA4^F/F^; SOD1^G93A^; homozygous deletion group, *n* = 11), EphA4^flox/WT^ × ChAT-Cre^KI/WT^ × SOD1^G93A^ mice (EphA4^F/W^; SOD1^G93A^; heterozygous deletion group, *n* = 8) and EphA4^flox/flox^ × ChAT-Cre^WT/WT^ × SOD1^G93A^ mice (EphA4^W/W^; SOD1^G93A^; normal expression group, *n* = 10). We also included EphA4^flox/flox^ × ChAT-Cre^KI/WT^ × SOD1 wild-type counterpart mice with conditional deletion of *EphA4* (EphA4^F/F^; WT, *n* = 11) and EphA4^flox/flox^ × ChAT-Cre^WT/WT^ × SOD1 wild-type counterpart mice with intact *EphA4* expression (EphA4^W/W^; WT, *n* = 9) as control groups. We ensured all experimental mice have the same genetic background, C57Bl/6J, and the control animals were on the same genetic background by using littermates of the SOD1^G93A^ mice, to cross with the EphA4^flox/flox^ and ChAT-Cre^KI/KI^. During the entire study, no mice in either the experimental or control groups showed the characteristic abnormal, kangaroo-like, hopping gait of *EphA4* germline deletion mutants^1^, indicating that specific postnatal deletion of EphA4 did not affect developmental axon guidance. All experimental animals were sacrificed when any of the following signs was observed, including loss of the righting reflex, greater than 20% of their highest body weight loss, or complete paralysis of any hind-limb rendering the animal incapable of reaching food and water, which was identical to that of the above mutEphA4-Fc treatment experiment. Mice in the homozygous deletion group (EphA4^F/F^; SOD1^G93A^) were produced according to a Mendelian distribution and their size and body weight were comparable to those of normal SOD1^G93A^ mice. Interestingly, no differences in body weight were observed between homozygous EphA4^F/F^; SOD1^G93A^ and EphA4^W/W^; SOD1^G93A^ groups; however, heterozygous EphA4^F/W^; SOD1^G93A^ mice were consistently heavier than SOD1^G93A^ mice in the other two groups (Fig. 2A) and the differences in body weight between EphA4^F/W^; SOD1^G93A^ and EphA4^W/W^; SOD1^G93A^ groups reached statistical significance at both 13 and 18 weeks of age (Two-way ANOVA, followed by Fisher’s LSD test, *F*_(60,660)_ = 8.838; week 13, *p* = 0.0383; week 18, *p* = 0.0177).

In line with the changes in body weight, we did not observe any differences in motor function between EphA4^F/F^; SOD1^G93A^ and EphA4^W/W^; SOD1^G93A^ groups, whereas heterozygous deletion of *EphA4* in SOD1^G93A^ mice showed improved functional performance during the disease course relative to the EphA4^W/W^; SOD1^G93A^ group.

Specifically, EphA4^F/F^; SOD1^G93A^ and EphA4^W/W^; SOD1^G93A^ mice showed steady decreases in rotarod test values until the end-point, while the decline in performance of EphA4^F/W^; SOD1^G93A^ mice was much slower than that of the other two groups before week 19, resulting in significant differences at week 17–19 compared with EphA4^W/W^; SOD1^G93A^ mice. After week 19, the performances of these three groups were similar (Two-way ANOVA, followed by Fisher’s LSD test, *F*_(60,660)_ = 13.8; week 17, *p* = 0.0012; week 18, *p* < 0.0001; week 19, *p* < 0.0001) (Fig. 2B). In the hind-limb grip strength test, overall trends for the three groups were similar, with a gradual increase in grip strength until week 7 that subsequently fell until the end-point. Importantly, hind-limb grip strength in EphA4^F/W^; SOD1^G93A^ mice was always higher than that in EphA4^W/W^; SOD1^G93A^ mice, and significant differences were reached at week 7, 8, 10, 18 and 19 (Two-way ANOVA, followed by Fisher’s LSD test, *F*_(60,660)_ = 25.94; week 7, *p* = 0.0028; week 8, *p* = 0.0018; week 10, *p* = 0.0181; week 18, *p* = 0.0500; week 19, *p* = 0.0467) (Fig. 2C).

During the same time interval, the body weights and functional performance of the control groups (EphA4^F/F^; WT and EphA4^W/W^; WT) were significantly higher than the three SOD1^G93A^ experimental groups. The body weights of the control groups gradually increased from week 5 to week 22. Although EphA4^W/W^; WT mice were generally heavier than EphA4^F/F^; WT after 12 weeks of age, the differences were not significant (Fig. 2A). In the rotarod test, EphA4^F/F^; WT and EphA4^W/W^; WT mice continued to perform at near optimal levels (Fig. 2B). Additionally, the hind-limb grip strength of EphA4^F/F^; WT and EphA4^W/W^; WT mice steadily increased with age (Fig. 2C). Like the changes in body weight, EphA4^W/W^; WT mice had a somewhat but not significantly greater hind-limb grip strength than EphA4^F/F^; WT mice (Fig. 2C).

Disease onsets occurred significantly later in EphA4^F/F^; SOD1^G93A^ and EphA4^F/W^; SOD1^G93A^ groups than in the EphA4^W/W^; SOD1^G93A^ group (EphA4^F/F^; SOD1^G93A^ = 115 ± 2.6 days; EphA4^F/W^; SOD1^G93A^ = 111.5 ± 3.74 days; EphA4^W/W^; SOD1^G93A^ = 98.5 ± 3.5 days; log-rank test; *p* = 0.0015) (Fig. 2D). However, the deletion of EphA4 did not substantially affect the median survival times of each group, which were 158, 155.5 and 156 days for EphA4^F/F^; SOD1^G93A^, EphA4^F/W^; SOD1^G93A^ and EphA4^W/W^; SOD1^G93A^ groups, respectively (EphA4^F/F^; SOD1^G93A^ = 141–167 days; EphA4^F/W^; SOD1^G93A^ = 143–175 days; EphA4^W/W^; SOD1^G93A^ = 140–163 days, log-rank test; *p* = 0.47).

**Figure 3:**
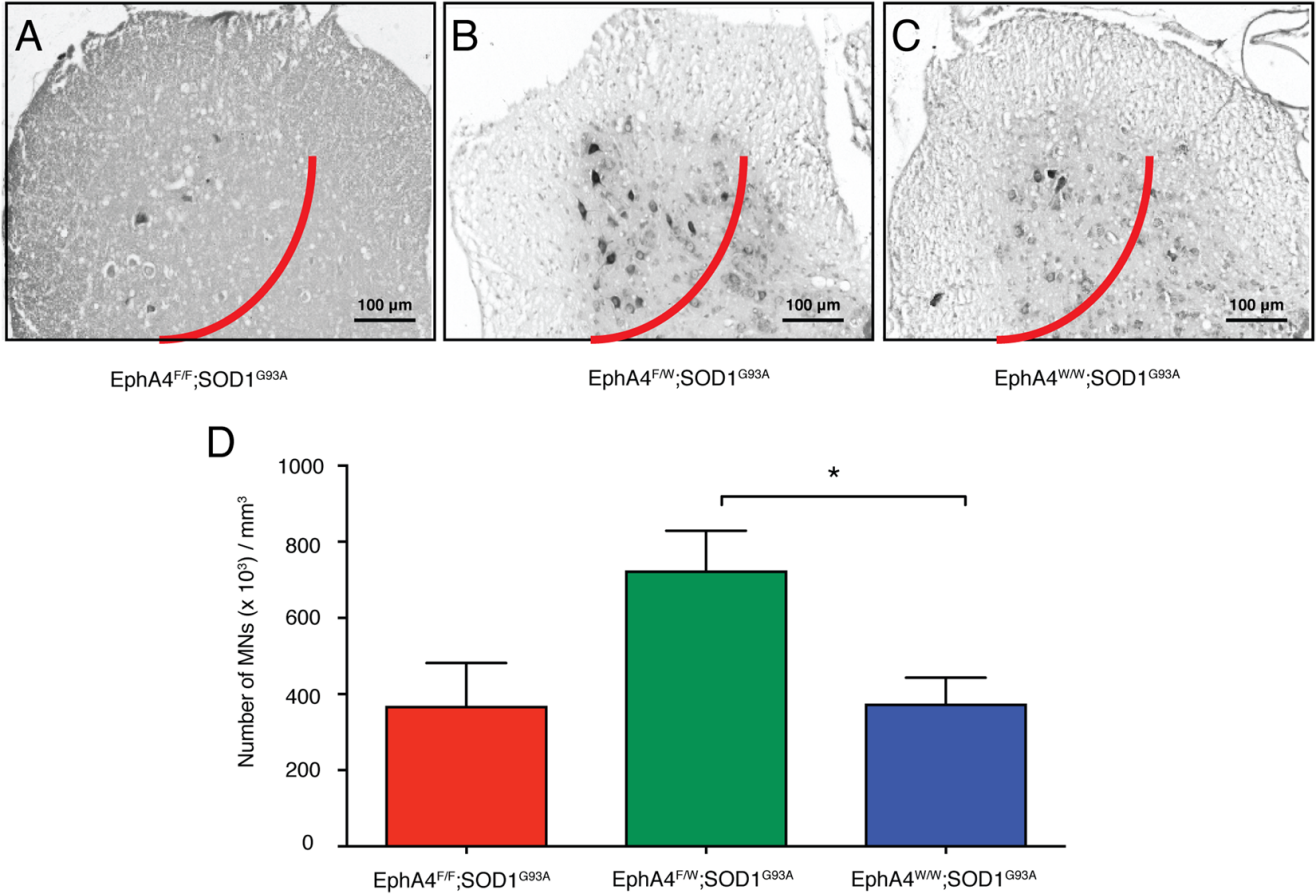
Heterozygous knockout of the EphA4 gene in SOD1^G93A^ mice prevents the death of motor neurons in the spinal cord. Representative images of motor neurons stained with thionine in the lumbar enlargement of the spinal cords (left of the red lines) of (**A**) EphA4^F/F^; SOD1^G93A^, (**B**) EphA4^F/W^; SOD1^G93A^ and (**C**) EphA4^W/W^; SOD1^G93A^ mice at 17 weeks of age. (**D**) The number of motor neuron cell bodies per mm^3^ of the spinal cord in these three groups. Data are expressed as mean ± SEM of n = 3–5 mice in each group, one-way ANOVA with Tukey’s multiple comparisons test, *p < 0.05.

We then examined the number of motor neurons in the lumbar enlargement of the spinal cord from EphA4^F/F^; SOD1^G93A^ (*n* = 3), EphA4^F/W^; SOD1^G93A^ (*n* = 5), and EphA4^W/W^; SOD1^G93A^ (*n* = 5) at 17 weeks of age. To count cells in an unbiased method, serial transverse sections (10 μm) of the lumbar enlargement were collected and motor neurons, which were identified by thionine staining, according to previously described^18,19^, were counted within the lateral motor column on both sides of the spinal cord in every fifth section, as shown by previously studies^19–22^. Approximately 100 sections per spinal cord per animal were counted. Total motor neuron counts were normalised by the volume of the tissues counted^19^, and data was expressed as number of cells per mm^3^. This method followed an unbiased stereology principal. Each animal was coded independently by different individual with random numbers to avoid sampling bias with codes revealed at the conclusion of analysis. Representative examples are shown in Fig. 3A–C. Spinal cords from EphA4^F/W^; SOD1^G93A^ mice contained significantly more motor neurons than EphA4^F/F^; SOD1^G93A^ and EphA4^W/W^; SOD1^G93A^ mice (One-way ANOVA, followed by *post hoc* Tukey’s test, *F*_(2,10)_ = 4.982; EphA4^F/F^; SOD1^G93A^ vs. EphA4^F/W^; SOD1^G93A^, *p* = 0.0756; EphA4^F/W^; SOD1^G93A^ vs. EphA4^W/W^; SOD1^G93A^, *p* = 0.0433) (Fig. 3D). We also examined the number of motor neuron in the spinal cord at the end-point among these three groups. However, no difference in the motor neuron number was observed (see Supplementary Fig. 2A). Similarly, the number of motor neurons in the spinal cord was same at the end-point between the mutEphA4-Fc-treated group and the vehicle control group (see Supplementary Fig. 2B).

**Figure 4:**
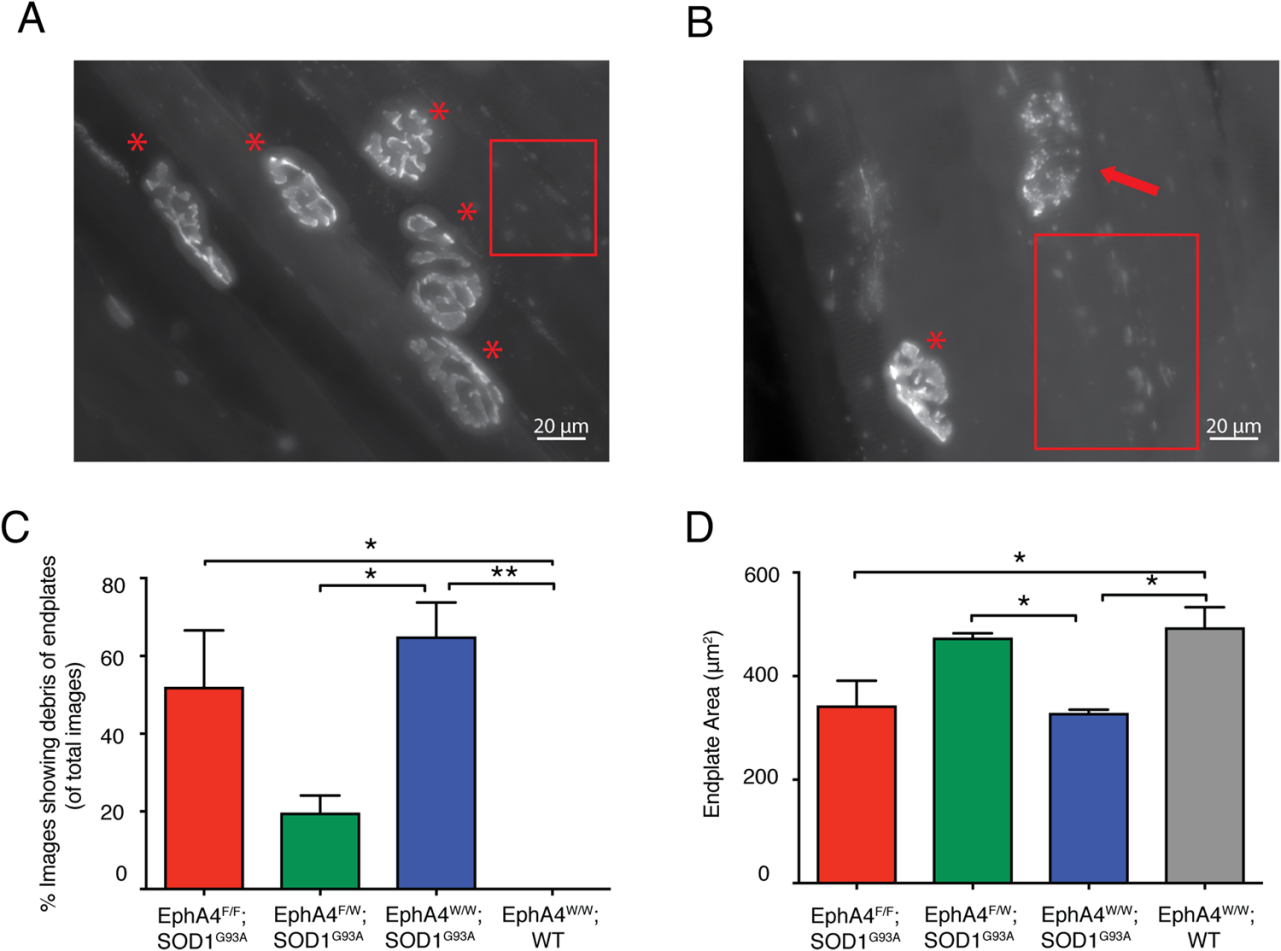
Heterozygous knockout of the EphA4 gene in SOD1^G93A^ mice maintain the better morphology of the post-synaptic endplates of NMJs in the TA muscle. Representative images of endplates of NMJs labelled with Alexa Fluor 555 α-BTX of TA muscles of (**A**) EphA4^F/W^; SOD1^G93A^ mice and (**B**) EphA4^F/F^; SOD1^G93A^ mice at 17 weeks of age. Images show maximum intensity projection of several endplates of NMJs. (**A**) “Pretzel-like” normal endplates are labelled with red asterisks, and (**B**) a fragmented endplate is marked with a red arrow. (**A**,**B**) Many debris of damaged endplates are observed in the red rectangles. (**C**) The percentage of images that show debris of damaged endplates surrounding the region of interesting endplates in total endplate images among EphA4^F/F^; SOD1^G93A^, EphA4^F/W^; SOD1^G93A^, EphA4^F/W^; SOD1^G93A^ and EphA4^W/W^; WT groups. EphA4^F/F^; SOD1^G93A^ mice and normal SOD1^G93A^ mice contain significant more images showing debris of endplates than that in EphA4^W/W^; WT mice. Furthermore, a significantly fewer images of EphA4^F/W^; SOD1^G93A^ mice show damaged post-synaptic endplates than that of EphA4^W/W^; SOD1^G93A^ mice. (**D**) The area of post-synaptic endplates of TA muscles of three transgenic mouse groups and the wildtype controls. The TA muscles of EphA4^F/W^; SOD1^G93A^ mice contain significantly larger endplates, compared to that of EphA4^F/F^; SOD1^G93A^ mice and EphA4^F/F^; SOD1^G93A^ mice, which are in a similar size with that in the EphA4^W/W^; WT controls. Data are expressed as mean ± SEM. n = 3 mice in each group, endplates images = 35–40 per mouse, NMJs = 40–45 per mouse, one-way ANOVA with Tukey’s multiple comparisons test, *p < 0.05, **p < 0.01.

We next investigated the morphology of neuromuscular junctions (NMJs) on whole mounts of tibialis anterior (TA) muscle from EphA4^F/F^; SOD1^G93A^, EphA4^F/W^; SOD1^G93A^, EphA4^W/W^; SOD1^G93A^ and EphA4^W/W^; WT at 17 weeks of age (n = 3 mice per each group, endplates images = 35–40 per mouse, NMJs = 40–45 per mouse). The TA muscles were incubated with Alexa Fluor 555-alpha bungarotoxin (α-BTX; Molecular Probes, Invitrogen, Eugene OR, USA) diluted at 1:1000 in phosphate buffered saline (PBS) followed by fixation with 2% paraformaldehyde (PFA)/PBS and then washed with PBS. The tissues were blocked with 2% bovine serum albumin (BSA), and 0.5% TritonX-100 (TX-100)/PBS for 1 hour followed by washing with 0.5% TX-100/PBS. The tissues were then incubated with the following primary antibodies overnight at 4 °C; mouse anti-synaptic vesicle protein 2 (SV2) (1:500; Developmental Studies Hybridoma Bank) and rabbit anti-neurofilament (1:500 dilution; Sigma-Aldrich, Sydney, Australia). The tissues were washed with 0.5% TX-100 prior to incubation with appropriate Alexa Fluor 488-conjugated secondary antibodies (1:1000 dilution; Invitrogen; Carlsbad, CA) for 3 hours at room temperature. Tissues were mounted with Prolong Gold anti-fade mounting medium, and imaged with Zeiss Axio-Imager microscope and Axio-Vision v4.8 software^23^. Z-stacks of unbiased en face endplates were acquired consistently with a 0.5 μm step at 40x/0.75 magnification, constant illumination levels (HXP 120 lamp), with identical exposure time, same photomultiplier gain levels and pinhole size between the different slides^24^. Maximum intensity projections were generated with Fiji v1.0. As shown in Fig. 4A,B, except normal “pretzel”-like endplates, many fragmented postsynaptic endplates of NMJs and debris of damaged endplates were observed in the TA muscles from three groups at 17 weeks of age, including EphA4^F/F^; SOD1^G93A^ mice, EphA4^F/W^; SOD1^G93A^ mice and EphA4^F/W^; SOD1^G93A^ mice. Interestingly, about 65% of endplate images of EphA4^W/W^; SOD1^G93A^ mice showed lots of debris of endplates out of regions of interesting endplates, the percentage of the EphA4^F/F^; SOD1^G93A^ group was 52%, but only about 20% of images of EphA4^F/W^; SOD1^G93A^ mice showed damaged endplates. As for the EphA4^W/W^; WT mice, all of the endplates of NMJs we observed showed normal “pretzel”-like architecture, and no image showed debris of endplates. Both homozygous deletion group and normal SOD1^G93A^ mice contained significantly more images showing debris of endplates, compared to that of EphA4^W/W^; WT controls. Moreover, the percentage of images showing debris of endplates in the EphA4^F/W^; SOD1^G93A^ mice was significantly fewer than that in normal SOD1^G93A^ mice. (One-way ANOVA, followed by *post hoc* Tukey’s test, *F*_(3,8)_ = 11.45; EphA4^F/F^; SOD1^G93A^ vs. EphA4^W/W^; WT, *p* = 0.0129; EphA4^W/W^; SOD1^G93A^ vs. EphA4^W/W^; WT, *p* = 0.0035; EphA4^F/W^; SOD1^G93A^ vs. EphA4^W/W^; SOD1^G93A^, *p* = 0.0267) (Fig. 4C). En face postsynaptic endplates from these images were measured for different parameters, including endplate area (the surrounding region of the endplates), and average staining intensity (the density of the endplates)^24^. The area of postsynaptic endplates of EphA4^F/W^; SOD1^G93A^ mice was larger than that in both EphA4^F/F^; SOD1^G93A^ and EphA4^W/W^; SOD1^G93A^ mice, which was similar with that in EphA4^W/W^; WT mice. The difference in the area of endplates between EphA4^F/W^; SOD1^G93A^ mice and normal SOD1^G93A^ mice were statistically significant (One-way ANOVA, followed by *post hoc* Tukey’s test, *F*_(3,8)_ = 7.543; EphA4^F/F^; SOD1^G93A^ = 343 ± 48.15 μm^2^; EphA4^F/W^; SOD1^G93A^ = 474.3 ± 8.299 μm^2^; EphA4^W/W^; SOD1^G93A^ = 328.8 ± 6.445 μm^2^; EphA4^W/W^; WT = 494.2 ± 38.88 μm^2^; EphA4^F/F^; SOD1^G93A^ vs. EphA4^W/W^; WT, *p* = 0.0376; EphA4^W/W^; SOD1^G93A^ vs. EphA4^W/W^; WT, *p* = 0.0242; EphA4^F/W^; SOD1^G93A^ vs. EphA4^W/W^; SOD1^G93A^, *p* = 0.0450) (Fig. 4D). However, the difference in average intensity of endplates among four groups was not statistically significance (EphA4^F/F^; SOD1^G93A^ = 76.28 ± 1.806 units; EphA4^F/W^; SOD1^G93A^ = 83.81 ± 15.98 units; EphA4^W/W^; SOD1^G93A^ = 71.31 ± 4.695 units; EphA4^W/W^; WT = 79.04 ± 9.179 units). These results suggest that the morphology of postsynaptic endplates in the TA muscles is maintained better in EphA4^F/W^; SOD1^G93A^ mice, compared to that in both EphA4^F/F^; SOD1^G93A^ and EphA4^W/W^; SOD1^G93A^ mice. Immunostaining of presynaptic nerve terminal from three groups showed that the presynaptic terminals were observed in the NMJs containing normal “pretzel”-like endplates, whereas no presynaptic components were localised with fragmented endplates. Furthermore, no significant change in innervation was observed in the NMJs containing normal “pretzel”-like endplates among EphA4^F/F^; SOD1^G93A^, EphA4^F/W^; SOD1^G93A^ mice and normal SOD1^G93A^ mice (n = 3 mice per each group, NMJs = 40–86 per genotypes, One-way ANOVA, followed by *post hoc* Tukey’s test, *F*_(2,6)_ = 0.1169; EphA4^F/F^; SOD1^G93A^ = 86.14 ± 3.2%; EphA4^F/W^; SOD1^G93A^ = 89.25 ± 7.1%; EphA4^W/W^; SOD1^G93A^ = 89.87 ± 6.5%). Therefore, by way of increase in the normal endplates, there were more presynaptic axonal terminals existing in the EphA4^F/W^; SOD1^G93A^ mice than that in the EphA4^F/F^; SOD1^G93A^, and normal SOD1^G93A^ mice.

### Data Availability Statement

All data involved in this project is available.

### Ethical Approval

All animal experiments were conducted in accordance with the Australian Code of Practice for the Care and Use of Animals for Scientific Purposes, including housing of animals and procedural guidelines. Animal breeding and experimental ethical approval was obtained from the University of Queensland Animal Ethics Committee. All methods applied in this project were in accordance with the relevant guidelines and regulation of the Queensland Brain Institute and University of Queensland.

## Discussion

This is the first demonstration that loss or inhibition of EphA4 increases motor neuron survival in an ALS mouse model, resulting in improved functional performance. One of the possible mechanisms underlying this effect is that inhibition of EphA4 might decrease activation of caspase-3 or caspase-like enzyme, as EphA4 has been shown to induce the death of NIH 3T3 cell lines *in vitro* by increasing caspase-3 or caspase-like enzyme activity^25,26^. Given that both neuroinflammation and neuronal excitotoxicity have been shown to play important roles in neuronal death and promote ALS progression^27–31^, it is plausible that inhibition of EphA4 function may decrease neuroinflammation^32–34^ and neuronal excitotoxicity induced by glutamate^35^. Further investigation is required to address these potential underlying mechanisms individually.

In the mouse lines used in this study, EphA4 depletion was restricted to ChAT-positive neurons, which are located in the basal forebrain, cortex, striatum, midbrain, brainstem and spinal cord^16,17,36^. Therefore, further investigation is required to determine which region is most critical. However, given that the upper and lower motor neurons that degenerate with ALS progression are predominantly localised to the motor cortex, brainstem and spinal cord^37^, the deletion of EphA4 in these regions is most likely to account for the improved functional performance compared with SOD1^G93A^ mice with normal EphA4 expression.

Previous studies have demonstrated that homozygous germline deletion of EphA4 in SOD1^G93A^ mice were born with low body weight and barely survived to adulthood ^6^. On the contrary, the present study showed that SOD1^G93A^ mice with homozygous deletion of EphA4 in ChAT-positive cells were generated on the basis of Mendelian distribution and went through adulthood like normal SOD1^G93A^ mice do. This is not surprising, given the vital role of EphA4 in the development CNS^1,4^. Surprisingly, our results showed that only SOD1^G93A^ mice with heterozygous deletion of EphA4 in ChAT-positive cells conferred a protective effect on motor neurons survival, whereas, homozygous deletion of EphA4 in SOD1^G93A^ mice was similar to normal SOD1^G93A^ controls. This result may have several possible explanations including that low levels of EphA4 activity may be necessary for the survival of motor neurons. However, it may also mean that the other possibility underlying this different in the motor neuron survival is compensatory changes in functions of other molecules involved in the maintenance of homeostasis in motor neurons. Such compensation may possibly result in motor neuron death equivalent to that observed in cells expressing normal levels of EphA4, thus resulting in no improvement in the homozygous EphA4 deleted mouse line compared with normal SOD1^G93A^ mice. In comparison, heterozygous deletion of EphA4 may be insufficient to induce compensatory mechanisms resulting in a reduced level of EphA4, which would maintain normal CNS function and protecting motor neurons from death in ALS progression. This is supported by reports that the expression of ephrin-B2, EphA2 and ephrin-A1 is increased in cerebellar Purkinje cells of EphA4 knockout mice, which likely compensate for the function of EphA4 in patterning the Purkinje cells compartment^38^. Moreover, members of the Eph/ephrin family are involved in regulation of cell death in the brain, such as ephrin-A1^39^, ephrin-A5, EphA7^40^ and EphB2^41^. Recently, Ling and colleagues reported that reduction of mRNA expression of EphA4 in the CNS by antisense oligonucleotides (ASO) did not result in significant improvement in functional performance^42^. Considering that only approximately 20% EphA4 protein was detected in the brain and no EphA4 protein expression was found in the spinal cord after the EphA4-ASO treatment, such low level of EphA4 expression might be insufficient to maintain normal survival of motor neurons, protect them from death and subsequently improve functional performance in ALS disease progression, just like our homozygous deletion of EphA4 in ChAT-positive cells in SOD1^G93A^ mice did. Therefore, further investigation is required to address to what extent levels of EphA4 expression is required for motor neuron survival, whether and how such compensation may result in the difference between the homozygous and heterozygous groups in the ALS model.

In addition, our results showed that the area of postsynaptic endplates in the TA muscles of heterozygous deletion of EphA4 in ChAT-positive cells in SOD1^G93A^ mice was similar with that in EphA4^W/W^; WT controls, which was significantly larger than that in the normal SOD1^G93A^ mice. Moreover, significantly fewer images showing damaged endplates of NMJs were observed in the heterozygous deletion group, compared to normal SOD1^G96A^ mice. In addition, more intact presynaptic nerve terminals were surviving in the heterozygous deletion group than other groups. These results suggest that the 50% deletion of EphA4 in ChAT-positive cells maintains the better postsynaptic architecture of the NMJs, which also likely contributes to the improvement in the functional performance of SOD1^G93A^ mice. This is likely to be a result of more motor neurons survival in the heterozygous deletion group, as NMJs maintenance strongly depends on the acetylcholine released from the presynaptic specialization that is the nerve terminal of motor neurons^43^. It would be interesting to examine both motor neuron loss in the spinal cord and morphology of NMJs in the TA muscles along the entire disease progression in this mouse model in the future. Of note, the expression of EphA4 has been observed in the postsynaptic apparatus^44^, the application of mutEphA4-Fc is likely to affect the morphology of endplates of NMJs to cooperatively contribute to its diminution of ALS progression.

Another point to be noted is that the heterozygous deletion group showed increased body weight, whereas the homozygous group did not. It has been reported that underweight women with ALS have a higher risk of death than those of normal weight, and men with higher body mass index (BMI) have a lower risk of dying of ALS than those with lower BMI^45^, while a high-energy diet has been reported to extend the survival of ALS mouse models by 20%^46^. Moreover, nutritional intervention aimed at increasing body weight has been mooted as a therapeutic strategy for ALS patients^47^. Thus, the higher body weight of the EphA4^F/W^; SOD1^G93A^ mice may also be associated with the improved functional performance of this group.

Importantly, we observed that mutEphA4-Fc treatment improved the functional performance of SOD1^G93A^ mice compared with the saline-treated control group. Comparison of the two mechanisms for reducing EphA4 expression reported here showed that the pharmacological treatment was at least as, if not more effective than heterozygous deletion of *EphA4* in motor neurons. Given that mutEphA4-Fc can be regarded as a “pan-ephrin blocker” due to its ability to bind to both A and B type ephrins^9^, the blockade of many EphA4-ephrin interactions also cooperatively slow ALS progression.

Given that mutEphA4-Fc ameliorated the symptoms of SOD1^G93A^ mice, delaying disease onset and extending survival to some extent, these effects imply that intraperitoneally administered mutEphA4-Fc is able to cross the blood–brain barrier (BBB) and the blood–spinal cord barrier (BSCB) to modify ALS progression, as indicated by the improved functional performance. This presumption is also supported by data showing that intraperitoneally injected EphA4-Fc significantly improved axonal regeneration and functional recovery following contusive spinal cord injury^3^. Moreover, studies of the BBB and BSCB in ALS patients and animals detected vascular leakage, swollen endothelial cells and degenerating capillary rupture in both early and end-stage disease^48–51^. The infiltration of inflammatory cells and impaired astrocytic end-feet are also involved in BBB and BSCB damage in ALS^52–56^. Thus, the BBB and BSCB in ALS animals is significantly compromised, allowing mutEphA4-Fc to enter the CNS and suggests that peripheral administration of mutant EphA4-Fc is a viable therapeutic option.

Noteworthy, the inhibition of EphA4 by either the mutEphA4-Fc treatment or 50% deletion in the ChAT-positive cells delayed ALS disease onset. A similar delayed effect on disease onset was also observed when the expression of EphA4 was reduced by the EphA4-ASO treatment^42^. However, our results showed that the EphA4 inhibition did not significantly extend median survival time of SOD1^G93A^ mice. Given that no increased motor neuron survival in the spinal cord was observed in either the mutEphA4-Fc-treated mice or EphA4^F/W^; SOD1^G93A^ mice at the end-point of both experiments, compared to their counterpart control groups. The inhibition of EphA4 is most likely to modify the early stage of ALS and maintain motor neuron alive, resulting in improvements in the functional performance during ALS disease progression; however, it is unlikely to alter longitudinal motor neuron death at the end of disease.

It should also be noted that all experiments here were conducted with SOD1^G93A^ mice or based on the SOD1^G93A^ genetic background, which have their limitations for modelling all forms of ALS, particularly TDP43 and C9orf72, and other major hallmarks of sporadic and familial ALS^57^. However, this result also suggests that ALS patients carrying mutant SOD1 are likely to have a better response to the mutEphA4-Fc treatment, which shed a light on the future clinical application. Of note, the mutEphA4-Fc treatment was administered to SOD1^G93A^ mice prior to disease onset. While it is not consistent with the potential clinical application in most case, it may be practical in familial ALS patients diagnosed with SOD1 mutation. Moreover, the early mutEphA4-Fc treatment is consistent with the report that EphA4 is likely to be more critical in early disease pathogenesis^58^. Therefore, earlier mutEphA4-Fc treatment is most likely to obtain a better clinical outcome for ALS patients.

In summary, the present study has demonstrated that partial inhibition of EphA4 significantly extends the time until disease onset and improves functional performance in an ALS disease model. It also suggests that EphA4 signalling contributes to motor neuron death and eventual ALS disease progression. Hence, reducing the downstream consequences of EphA4 activation through specific inhibitors is a promising approach for therapeutic intervention to slow disease progression in ALS.

## Electronic supplementary material

Supplementary Information
