# Supplementary material for: Decreased signalling of EphA4 improves functional performance and motor neuron survival in the SOD1G93A ALS mouse model
Source: Sci Rep. 2018 Jul 30;8:11393. doi: 10.1038/s41598-018-29845-1 (PMC6065374; doi:10.1038/s41598-018-29845-1)
Supplement: Supplementary file 1 — Supplementary Information [file 41598_2018_29845_MOESM1_ESM.pdf]

Decreased signalling of EphA4 improves functional performance and motor neuron survival in the SOD1G93A ALS mouse model

Zhao J[1], Cooper LT[1,2], Boyd AW[2], Bartlett PF[1*].

1. Queensland Brain Institute, University of Queensland, Brisbane, Queensland, Australia.
2. Queensland Institute of Medical Research, Brisbane, Queensland, Australia.

*Corresponding author
Perry F. Bartlett,
Tel: (61 7) 336 51603
Email: p.bartlett@uq.edu.au

Supplementary Figure 1

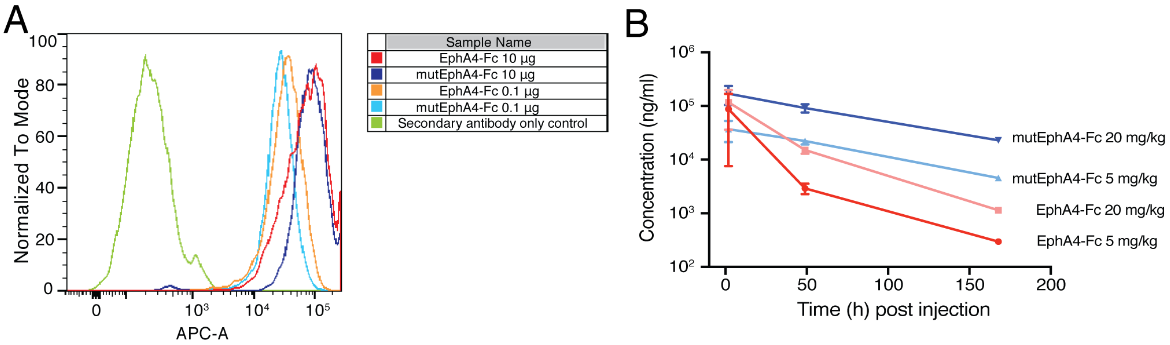

Supplemental Figure 1. Ligand-binding and pharmacokinetic analyses of the mutant mouse EphA4-Fc compared with the wild type protein. (A) Flow cytometric analysis of binding of the modified and wild type EphA4-Fc to an ephrin-A5-expressing CHO cell line at concentrations of 0.1 µg/ml and 10 µg/ml. The ephrin-A5-expressing CHO cell line was incubated with either mutEphA4-Fc or wild-type proteins (0.1 µg/ml and 10 µg/ml) for 1 h at 4 °C, washed twice with 5% (v/v) FCS in PBS prior to staining for 30 min at 4 °C with conjugated AlexaFluor 488 goat anti-human IgG (Invitrogen, Carlsbad, CA, USA) at 1:400 dilution. Following three washes in 5% (v/v) FCS in PBS, cells were analysed on a BD LSR Fortessa flow cytometer using FACS DIVA software. (B) Sandwich ELISA of mouse serum to detect mutant and wild-type EphA4-Fc clearance in mice. Sixteen C57BL/6 mice (8 weeks of age) were randomly divided into four groups (four mice per group), with mutEphA4-Fc or wild-type protein at 5 mg/kg or 20 mg/kg administered by intraperitoneal injection. Serum was collected at 0.5 h, 48 h, and 7 days post injection by retro-orbital bleeds and collection of 150–200 µl of blood into a Microvette blood collection tube. Following incubation for 30–45 min in an upright position at room temperature, tubes were centrifuged at 11,000 rpm for 10 min prior to serum removal. Serum was stored at −80°C prior to ELISA analysis. All animal experiments were conducted in accordance with the Australian Code of Practice for the Care and Use of Animals for Scientific Purposes, including housing of animals and procedural guidelines. Animal breeding and experimental ethical approval was obtained from the University of Queensland Animal Ethics Committee.

Supplementary Figure 2

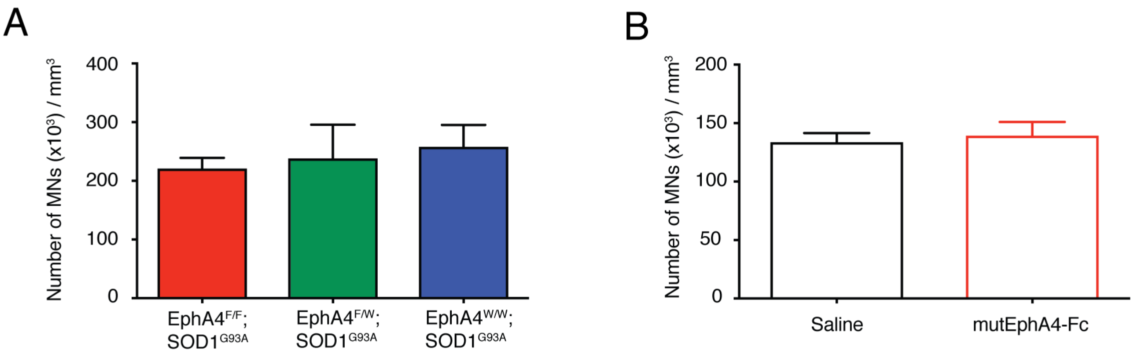

Supplemental Figure 2. The EphA4 inhibition does not affect the motor neuron survival at the end-point of ALS disease. The number of motor neuron cell bodies per mm^3 of the spinal cord in the transgenic mouse line experiment (A) and the mutEphA4-Fc-treatment experiment (B). No difference in the survival of motor neuron in the spinal cord is observed in either the $EphA4^{F/W}$; $SOD1^{G93A}$ mice or mutEphA4-Fc-treated mice at the end-point of both experiments, compared to their counterpart control groups. Data are expressed as mean ± SEM. n = 3 mice per group.
